# Prevalence and factors associated with sexual and reproductive health services use among reproductive age women with disabilities: a community based cross-sectional study

**DOI:** 10.1186/s12905-023-02373-5

**Published:** 2023-05-02

**Authors:** Bayew Kelkay Rade, Animut Tagele Tamiru, Getie Lake Aynalem, Eden Bishaw Taye, Mamaru Melkie, Alamirew Abera, Endeshaw Admassu Cherkos, Mengstu Melkamu Asaye

**Affiliations:** 1 Department of General Midwifery, School of Midwifery, College of Medicine and Health Sciences Comprehensive Specialized Hospital, University of Gondar, Gondar, Ethiopia; 2 Department of Clinical Midwifery, School of Midwifery, College of Medicine and Health Sciences Comprehensive Specialized Hospital, University of Gondar, Gondar, Ethiopia; 3 Department of Special Need, College of Social Sciences, University of Gondar, Gondar, Ethiopia; 4 Labour and Social Affairs Office, Gondar, Ethiopia; 5 Department of Women’s and Family Health, School of Midwifery, College of Medicine and Health Sciences Comprehensive Specialized Hospital, University of Gondar, Gondar, Ethiopia

**Keywords:** Disabilities, Reproductive-aged, SRH, Uptake, Women

## Abstract

**Background:**

According to International Convention on the Right of Person with Disabilities (CRPD), all nations should discern Sexual and Reproductive Health (SRH) as human rights and needs of all people living with disabilities. Women and girls with disabilities are highly vulnerable to SRH disparities including unintended pregnancy, acquiring sexual transmitted infections and unsafe abortion. Little has known about SRH service uptake and influencing factors among reproductive aged women living with disabilities.

**Methods:**

A community-based cross-sectional study was conducted from January 1–30, 2021, the central Gondar zone selected districts. A total of 535 reproductive-age (18–49 years) women with disabilities had been interviewed through face-to-face using structured questionnaire. Multistage cluster sampling method was applied. A binary logistic regression model was computed to look the relationship between independent variables and uptake of SRH, and p-value < 0.05 was a cut-off point to declare statistical significance.

**Results:**

A total of 33.27% (178/535) women with disabilities used at least one SRH service in the last twelve months preceding the survey. Those who had three or more children [AOR = 4.85; 95% CI (1.24–9.71)], autonomy to visit health care facilities [AOR = 3.30; 95% CI (1.45–6.92)], lived with sexual partner [AOR = 9.2; 95% CI (2.84–13.60)], subjected to radio/television in daily bases [AOR = 5.9; 95% CI (1.26–13.04)], autonomy to visit friends and relatives [AOR = 3.95; 95% CI (1.28–12.17)], had a discussion with family members about sexual and reproductive health [AOR = 9.36; 95% CI (3.44–17.47)], and engaged in sexual activity after the age of 18 years [AOR = 7.2; 95% CI (2.51–14.45)] were important predictors for service uptake.

**Conclusions:**

Only one in three reproductive age women with disabilities used at least one SRH service. These findings suggest that accessing information through mainstream media exposure, having full autonomous to visit friends and families, open discussion with family members, live with sexual partner, having optimal family size and starting sexual act at the recommended age improve the uptake of SRH services. Therefore, the stakeholders (both governmental and non-governmental) need to make efforts to increase the uptake of SRH services.

## Background

The World Health Organization (WHO) defined disability in many ways but generally refers to any casualty that prevents an individual from living their normal life. It includes people who are blind, deaf, physically disabled, intellectually impaired, or disabilities related to mental health. Disability is also explained as difficulties in any or all three areas of functioning: impairment, activity limitation and participation restriction [1, 2]. Around 15% of the world’s population or estimated more than a billion people live with disabilities, of whom 200 million experience considerable difficulties in functioning in daily life, and approximately 80% are living in developing countries [3]. Globally, there is a significant difference in the prevalence of disability between men and women: the male disability prevalence rate is 12% while the female is 19.2% [3, 4]. The World Bank and WHO estimated that there are about 15 million people living with disabilities in Ethiopia, standing for 17.6% of the population, and 14.0% in the Amhara region. However, this figure is agreed to be extremely low by different actors working in the area [5].

Disability is a human right issue; because people with disability experience inequalities in access to health care and education and subjected to violation of dignities like violence and abuse [6, 7]. Visually impaired teenagers are not well informed about prevention mechanism of unplanned pregnancy and sexually transmitted infections when they engage in pre-marital sexual acts [8]. Since Sexual and Reproductive Health (SRH) is being a significant part of health for sustainable development [9], many resource-limited countries have focused and set prevention strategies on the practice of unsafe sex, abortions, sexual transmitted infections including Human Immunodeficiency Virus (HIV), gender-based violence and other risk factors for adverse pregnancy outcomes [10, 11]. A population-based study conducted in Canada, Ontario revealed that, when women with disabilities have low accessibility and utilization of SRH programs and information may have poor preconception health [12]. The evidence from Northern Ireland has shown that family planning services do not adequately meet the needs of disabled clients because clinics are inaccessible in terms of physical access, informational exposure/access, and service provision. Social perspective on disabled people thought like they are asexual and not need to avail of reproductive health services including family planning [13]. In addition, SRH services are often inaccessible because of many reasons including stigma and discrimination, physical barriers, lack of accessible information and communication materials, health care providers’ negative attitudes and lack of disability related clinical services [14]. Qualitative study among women with hearing impairment (deaf) showed that inconsistent communication access and difficult in accessing health information were the barriers to get best pregnancy related care services [15]. Health providers who often assume disabled women are not sexually active, do not screen them for sexually transmitted infections (STIs), and do not provide contraceptives services and information about STIs including HIV. This exclusion has been found to be especially prevalent in many developing countries [14, 16]. United Nations General Assembly Convention article 25 stated that persons with disabilities have equal rights to SRH with nondisabled. This is important to achieve a Sustainable Development Goal (SDG) and create a truly inclusive society. However, the full number of SRH issues for women with disabilities is not yet clearly written, and highly unmet [17].

The SDG has striven to ensure ‘no one is left behind’ by promoting a stronger focus on disability. Accessing sexual and reproductive health services and reproductive rights for all persons with disabilities (targets 3.7 and 5.6) have been among the agendas of United Nation (UN) as SDG which could be achieved by 2030. Women with disabilities have the same SRH needs as women without disabilities. People with disabilities are among the most marginalized and disadvantaged by the community, and even by their family. They face physical, social, and attitudinal barriers in taking part as equal members of society and accessing community resources like health facilities in every part of their life, predominantly SRH services. However, few studies have been done on the real problems/gaps on SRH related issues. Thus, this study aimed to determine the prevalence of SRH service use and identify associated factors among reproductive age women with disabilities in Northwest Ethiopia.

## Methods

### Study setting, design and period

Community based cross-sectional study design was employed in Central Gondar zone of Amhara regional state which is found 748 Km to the Northwest of Addis Ababa, the capital city of Ethiopia. This zone has 15 woredas (districts) and one administrative town. According to Central Gondar zone reports related to risk exposure community of the year 2019/20 has shown that 5624 people lived with disabilities, and of these 2661 were females. Mi’irab Belesa, Gondar Zuria and Tach Armachiho were the three leading woredas reported high number of females living with disabilities [18]. The data was collected from January 1, 2021, to January 30, 2021.

### Source and study population

All visual and hearing impaired, and physical disabled women in the age range of 18 to 49 years in Central Gondar Zone were source population and those who lived in selected districts were study populations.

### Inclusion and exclusion criteria/s

Those reproductive age women (18–49 years) who had hearing and visual impairment, and physically disabled were included while seriously ill at the time of data collection were excluded. Interviewing intellectually/developmentally disabled people is challenging due to their limited communication abilities and difficulty in sustaining attention and concentration. Thus, the process requires excessive cost, time and experts.

### Sample size determination, sampling technique and procedure

To estimate the sample size of this study, single population proportion formula has used, where P = proportion of family planning utilization among reproductive aged disabled women in Arbaminch, Ethiopia was 33.7% [19], 95% confidence interval, 5% marginal error, considering 10% non-response rate and 1.5 design effect. Then, the final sample size with this assumption was 567. A multi-stage cluster sampling technique was applied. Clusters were chosen at random and every disabled woman in selected cluster was sampled.

Seven (7) woredas had been selected by lottery method and the total sampled participants were allocated proportionally based on the previous year’s number of disabilities reported by Central Gondar zone, social and labor office. Alefa, Aykel, Gondar Zuria, Misirak Dembiya, Chilga Kutir 1, Mi’irab Belesa, and Wegera woreda were selected. Again, the distributed sample size was distributed proportionally for each Kebele (smallest administrative unit in Ethiopia) and Gotts (governmental structure below kebele level in rural administrative structure which is similar with “Ketena” in urban administrative structure). All vision, hearing and physically disabled individuals aged 18–49 years in the households from selected areas had been included to this study.

### Study variables

Utilization of SRH service was the outcome variable, whereas, **background characteristics;** age, marital status, religion, occupation, educational level, partner occupation, educational level of spouse, residence, currently where she lives, family size, with whom she lives, **forms of disability, membership, and decision- making characteristic**s; membership to disability association, availability of support, living situation, exposure to medias, forms of disability and autonomy to visit health facilities, families, and other relatives, and **reproductive health history, service information and access related variables** like; age of menarche, ever have sex, age when have first sexual intercourse, sources of information about where to use and access SRH services source of information, and materials accessible in appropriate format were the explanatory variables.

### Operational definition

#### Utilization of SRH services

Appropriate package of one or more of SRH services (modern family planning, maternal care, cervical cancer screening, HIV/AIDS testing and other STIs screening, abortion/post abortion care) can be obtained by the women from pharmacy, health institutions (private or governmental), community-based distribution (rarely practiced in this study setting) or some other sources. Women who used at least one SRH service from the list five components considers as used the service. The question was forwarding like “Have you used at least one SRH services from the five components in the last 12 months preceding to the study?”, which was self-reported, and the list of the services had been validated by reminding the components [20, 21].

### Data collection tools and procedures

The women with disability had been screened from general population using rapid assessment disability (RAD) tool which was adapted from Washington Group for disability statistics to identify disabled women [22]. This tool includes a small number of questions and is recommended for use in community surveys because of its simplicity to overcome the practical and conceptual difficulties in measuring disability. Then, those identified vision, hearing or physically disabled women were further interviewed about SRH service utilization and associated factors. Some steps were involved in developing data collection tool (questionnaire). These were; setting the research aim, defining the target participants, deciding the research method to reach out the participants, content the questionnaire need to be included, and develop the questions in meaningful order and format. The tool was adapted from previous literatures [23–30]. These interviewers administer paper questionnaire used to gather/collect the primary data from the study participants. Since some questions were sensitive to ask in the study setting culture, interviewers were matched for gender. Interview was taking place in locations with privacy, such as closed rooms or quiet places in the house/institution. Fourteen (14) data collectors and 7 supervisors were recruited. Data collection was facilitated by experts in special need education and who had adequate prior experience of data collection in similar surveys. Additionally, professional sign language interpreters were involved. Both data collectors and supervisors had been trained for one day in interviewing techniques, purpose of the study, importance of privacy, and how to use questionnaires with practical demonstration.

### Data analysis and presentation

The collected data checked for completeness, coded, and then entered into Epi Data version 3.1 software and exported to SPSS (Statistical Package for Social Sciences) version 21 Software for analysis. Data organized and presented using tables, graphs, charts. Bivariable and multivariable logistic regression were performed to find statistically significant variables using a cut-off p < 0.2 in the bivariable analysis to identify candidate variables for multivariable logistic regression [31–33]. Adjusted odds ratio with 95% confidence interval used to declare statistically significant variables based on p < 0.05 in the multivariable logistic regression model. The Hosmer-Lemeshow test was performed to check the goodness of fit test and the decision was made at p > 0.05.

### Data quality management

The questionnaire was adapted from previous literatures conducted in various parts of the world. There was detailed communication with senior researchers, and experts in reproductive health and special need about the entire paper and check its theoretical validity, and their comments were considered. The questionnaire was translated from English to local language (Amharic) via forward and backward translation by experts in both languages. Besides, intensive training was provided to all interviewers and supervisors. Sign language experts took part in data collection during the interview of deaf participants. The questionnaire was pre-tested before the start of actual data collection on 5% (29 study subjects) of the total required sample size in Gondar city which was not selected as study area. Filled out questionnaires have been checked for completeness and its consistency every night at the time of data collection and incomplete questionnaires were sent back to the data collector for check-up under supervision. Based on the pretest results; clarity, wording, logical sequence, and skip patterns of the questions were amended.

## Results

### Background related characteristics

A total of 535 disabled reproductive age women were participated in this study with a response rate of 94.35%. More than two-fifths (42.1%) of the study participants were found in the age range of 35–49 years, and the mean age of them was 31.46 years (SD ± 8.93). More than half (54.6%) of the study participants were unable to read and write, and more than three-fourths.

(87.7%) of the study participants were orthodox religion followers. Most of the study participants (362/535) lived in the rural part of the study area. Regarding the employment and marital status; only 19 (3.6%) disabled participants were governmental employee and 129 (24.1) were married. Of those who were married, more than one-third (34.05%) of their sexual partners were unable to read and write, and half (49.72%) of their partner were farmers. Three-hundred nineteen (59.6%) study participants had three or more household members, and 45% (241/535) of the participants had no live child (Table 1).

**Table 1:** Background characteristics of reproductive-age women with disabilities in Northwest Ethiopia, 2021 (N = 535)

Variables	Category	Frequency	Percent (%)
Age in years	18–24	176	32.9
	25–34	134	25.0
	35–49	225	42.1
Educational level	Unable to read and write	292	54.6
	Able to read and/or write	49	9.2
	Elementary (1–8 grades)	107	20.0
	High school (9–12 grades)	50	9.3
	College and above	37	6.9
Religion	Orthodox	469	87.7
	Muslim	66	12.3
Resident	Urban	173	32.3
	Rural	362	67.7
Current employment	Housewife	177	33.1
	Governmental Employee	19	3.6
	Merchant/Private business	77	14.4
	Regularly beg from others	87	16.3
	Student	105	19.6
	Get supported from family members	48	9
	Daily laborer	22	4.1
Current sexual relationship status	Married	129	24.1
	Cohabitant or boyfriend	56	10.5
	Widowed	116	21.7
	Single	32	6.0
	Divorced	202	37.8
Sexual partner educational level (N = 185)	Unable to read and write	63	34.05
	Able to read and/or write	43	23.24
	Elementary (1–8 grades)	26	14.05
	High school (9–12 grades)	20	10.81
	College and above	33	17.85
Sexual partner occupation (N = 185)	Farmer	92	49.72
	Merchant/private business	29	15.68
	Governmental employee	23	12.44
	Student	24	13
	Daily laborer	17	9.16
Household size	Fewer than three	216	40.4
	Greater or equal to three	319	59.6
Number of alive children (birthed, adopted and stepchildren)	No child	241	45.0
	one child	93	17.4
	two children	75	14.0
	Three and above	126	23.6

### Forms of disability, membership, and decision-making related characteristics

From the total 535 disabled participants, 237(44.3%) of them were physically impaired followed by visual impairment (35%).

Only one-fifth of the study participants knew organizations deal with disabilities around where they live, and one-fourth (26.7%) of the participants were membership in any community group/s. More than half (57.6%) of the study participants were visiting health facilities for any care services without seeking others’ permission. Beside to this, nearly two-third (64.9%) of disabled women had no autonomy to visit their friends and relatives (decision making and freedom to move out of the house), and 198 (37.0%) were live alone. More than half (54.2%) of the study participants lived in their own home. Only 72 (13.5%) of the study participants were exposed to radio/television every day, and 234 (43.7%) participants decided alone when they wanted to do something (Table 2).

**Table 2:** Frequency distribution of participants by their forms of disabilities, membership and decision-making related variables in Northwest Ethiopia, 2021(N = 535)

Variables	Category	Frequency	Percent (%)
Time when disability occurred	Since birth	105	19.6
	Childhood (birth to 18 years)	259	48.4
	Later (after 18 years of age)	98	18.3
	I do not remember	73	13.6
Organization/s present around that work/s on people with disabilities empowerment	Yes	115	21.5
	No	420	78.5
Membership any community group/s	Yes	143	26.7
	No	392	73.3
Membership to any disability association/federation	Yes	73	13.6
	No	462	86.4
Autonomy to visits health facilities (decision making and freedom to move out of the house)	Yes	308	57.6
	No	227	42.4
Autonomy to visit friends and relatives (decision making and freedom to move out of the house)	Yes	188	35.1
	No	347	64.9
Living situation (with whom they live)	With parents	139	26.0
	With partner	131	24.5
	Alone in my home	198	37.0
	Live with children	28	5.3
	Live with other else	39	7.2
Currently live at	My own home	290	54.2
	Rented house	214	40.0
	Institution based	12	2.2
	Street based	19	3.6
Exposure to Radio/Television	Never	249	46.5
	Rarely	158	29.5
	Sometimes (2–3 days/week)	56	10.5
	Almost every day	72	13.5
Decision maker to do something (on all issues)	Me (herself)	234	43.7
	Husband/boyfriend	37	6.9
	Joint	103	19.3
	Other family members (caregivers)	161	30.1

### Reproductive health history, service information and access related variables

Almost half (49.9%) of the study participants experienced the first menstruation when they were in the age range of 15–18 years. One-hundred fifty-three (28.6%) of the participants never had sexual contact history, and 15.3% (82/535) were pregnant before the age of 18 years. The majority (96.8%) of respondents visited governmental health facilities when they needed medical assistance, and 29.2% (156/535) participants said that getting reproductive care service is exceedingly difficult. Half (49.9%) of study participants also mentioned, there was problem in accessing information related to SRH services, and two-thirds (67.5%) of the participant were not being recommended to be tested for HIV/AIDS or/and pregnancy and screened for cervical cancer. Almost four-fifths (78.3%) of the study participants replied that materials are accessible in proper format for people living with disabilities at health facilities (Table 3).

**Table 3:** Reproductive/sexual health history, service information and access related variables of participants in central Gondar, Northwest Ethiopia, 2021 (N = 535)

Variable	Category	Frequency	Percent (%)
Age of menarche	Before 15 years of age	140	26.2
	15–18 years of age	267	49.9
	Do not know	126	23.6
	Never seen	2	0.4
First sexual intercourse	Before 15 years of age	34	6.4
	15–18 years of age	143	26.7
	After 18 years of age	137	25.6
	Had never	153	28.6
	Do not remember	68	12.7
Age of first pregnancy	Below 15 years of age	6	1.1
	15–17 years of age	76	14.2
	Above or equal to 18 years	160	29.9
	Do not remember	116	21.7
	Never pregnant	177	33.1
Place where she seeks care when she sick	Private	13	2.4
	Governmental	518	96.8
	Other	4	0.7
Place where SRH care service most frequently accessed	Private	6	1.1
	Governmental	354	66.2
	Pharmacy	1	0.2
	Never used	174	32.5
Ease of getting reproductive care services	Very difficult	156	29.2
	Somewhat difficult	152	28.4
	Not difficult	185	34.6
	I do not know	42	7.9
Problem to get information related to SRH services	Problem	267	49.9
	No problem	259	48.4
	I do not know	9	1.7
Know where HIV/AIDS or/and pregnancy testing, and cervical cancer screening are provided	Yes	182	34.0
	No	353	66.0
Materials/equipment are available in proper format for people with disabilities in health facilities	Yes	4	0.7
	No	112	20.9
	I do not know	419	78.3

**Abbreviations:** HIV/AIDS, Human immunodeficiency virus/acquired immune deficiency syndrome; and SRH, sexual and reproductive health

### SRH service utilization and awareness about the services

**Fig. 1:**
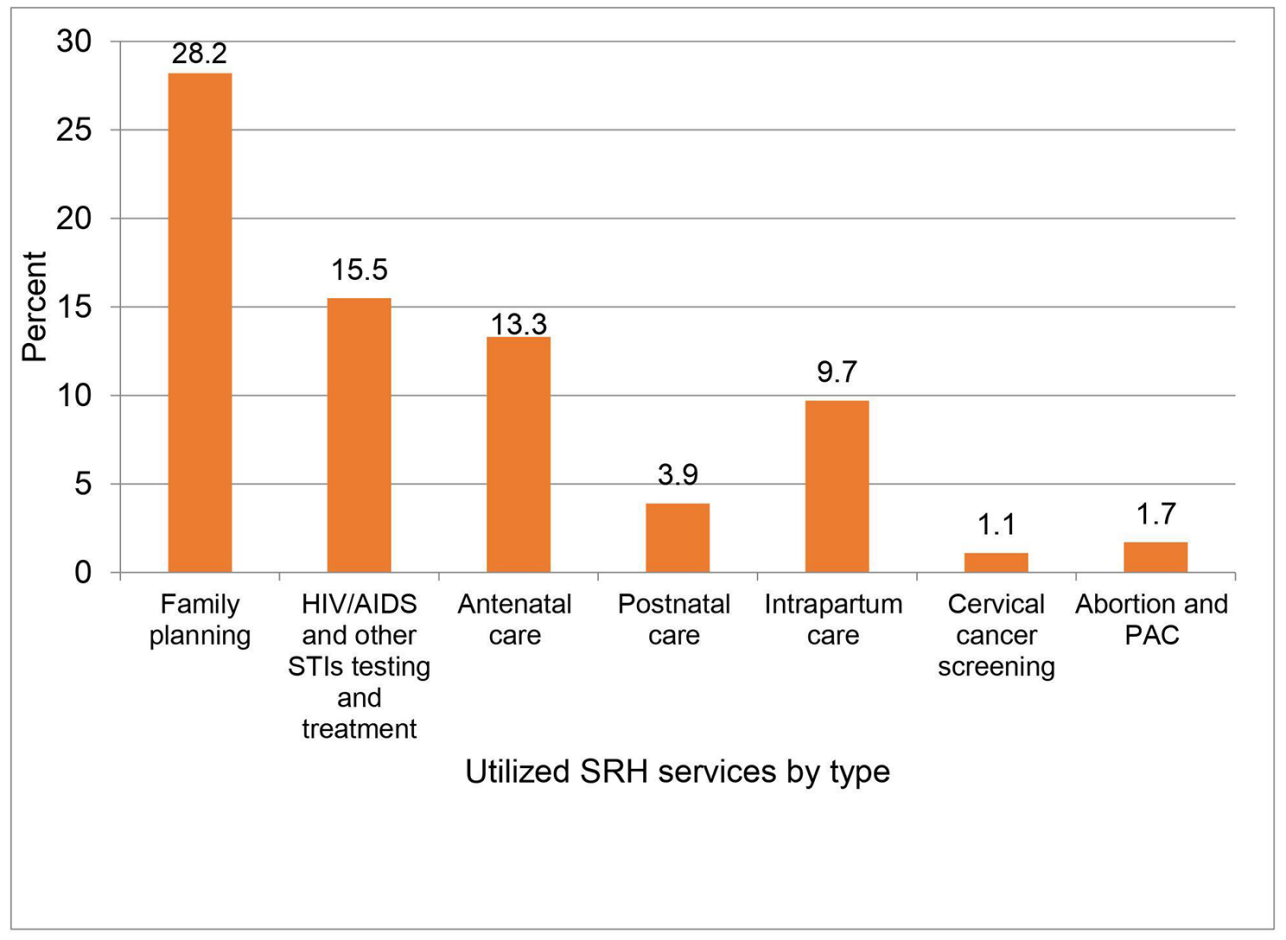
Types of SRH services used by reproductive-age women with disabilities in the last 12 months in Northwest Ethiopia, 2021 Abbreviations: HIV/AIDS, Human immunodeficiency virus/acquired immune deficiency syndrome; PAC, post-abortion care; and SRH, sexual and reproductive health.

Of the total study participants, 33.3% (178/535) utilized at least one sexual and reproductive health service in the last 12 months, and 151(28.2%) of them used family planning which is one of the components of SRH service (Fig. 1).

From those who utilized family planning, 108 (20.2%) of them used injectable (Depo-Provera) contraceptive method, and 108 (20.2%) participants preferred females as service provider (Table 4).

**Table 4:** SRH service utilization and awareness related factors among disabled reproductive aged women, Northwest Ethiopia, 2021(N = 535)

Variable	Category	Frequency	Percent (%)
Have ever heard about at least one SRH service?	Yes	424	79.3
	No	111	20.7
If “yes”, about what she heard? # Multiple answer was possible	Family planning	395	73.8
	HIV/AIDS and other STIs testing and treatment	286	53.5
	Cervical cancer screening	43	8.0
	Maternal care (ANC, IP & PNC)	245	45.8
	Abortion and PAC	31	5.8
The most common source of information	Mainstream media (TV or radio)	99	18.5
	Health professionals	270	50.5
	Associations	33	6.2
	Training	4	0.7
	Other	18	3.4
Discussed about SRH services within a family member	Yes	145	27.1
	No	390	72.9
Types of family planning used by the participants (n = 151)	Oral contraceptive pills	14	2.6
	Injectable	108	20.2
	Implanon	29	5.4
Preference of service provider’s sex for those who used the service (N = 178)	Male	17	3.2
	Female	108	20.2
	Anybody	53	9.9

**Note:** Other; neighbors, family members

**Abbreviations:** ANC, antenatal care; IP, intrapartum care; PAC, post-abortion care; PNC, postnatal care; and TV, television

### Factors associated with SRH service utilization 

A total of thirteen (13) independent variables were identified as factors in bivariate regression at p-value less than 0.2, and after controlling cofounders, seven (7) factors were found to have significant association with SRH service utilization in multivariate logistic regression at p-value less than 0.05. These were; age of first sexual activity, those who discussed about SRH services with family, exposure to radio/television, living with partner, number of live children, autonomy to visit health facilities, and able to visit friends and relatives without keeping any permission (Table 5).

**Table 5:** Regression table that shows the factors associated with SRH service/s uptake of reproductive age disabled women in Northwest Ethiopia, 2021

Variables	Uptake of SRH service/s		COR (95% CI)	AOR (95% CI)
	No (%)	Yes (%)		
Age in years				
18–24	138(78.4)	38(21.6)	1.00	1.00
25–34	85(63.40	49(36.6)	2.09 (1.267–3.46)	2.64 (0.82–17.40)
35–49	134(59.6)	91(40.4)	2.46 (1.58–3.86)	1.09 (0.14–8.73)
Educational level				
Unable to read and write	208(71.2)	84(28.8)	1.00	1.00
Able to read and/or write	29 (52.9)	20(40.8)	1.71(0.92–3.19)	1.41(0.47–9.83)
Elementary (1–8 grades)	77 (72.0)	30(28.0)	0.96(0.60–1.58)	0.44 (0.16–3.23)
Highschool (9–12 grades)	22(44.0)	28(56.0)	3.15(1.71–5.82)	2.01(0.22–18.69)
College and above	21(56.8)	16(43.2)	1.89(0.94–3.80)	0.38 (0.16–2.33)
Employment/occupation				
Housewife	62(35.0)	115(65.0)	1.00	1.00
Governmental Employee	12(63.2)	7(36.8)	0.31 (0.12–0.84)	0.02 (0.008–1.13)
Merchant/Personalbusiness	60(77.9)	17(22.1)	0.15 (0.10–0.28)	0.01 (0.006–1.06)
Regularly beg from others	70(80.5)	17(15.5)	0.13 (0.12–0.24)	0.33 (0.07–1.71)
Student	83(79.0)	22(11.0)	0.14 (0.10–0.25)	0.29 (0.08–1.10)
Others	64 (91.4)	6(8.6)	0.05 (0.02–0.84)	0.001(0.002–1.01)
Sexual relationship status				
Married	28(21.7)	101(78.3)	1.00	1.00
Cohabitant or boyfriend	24(42.9)	32(57.1)	0.37(0.19–0.73)	0.42 (0.10–1.75)
Widowed	106(91.4)	10(8.6)	0.03(0.01–0.06)	0.04 (0.02–1.25)
Single	17(53.1)	15(46.9)	0.24(0.11–0.55)	0.21 (0.03–1.34)
Divorced	182(90.1)	20(9.9)	0.03(0.02–0.06)	0.23 (0.12–1.36)
Household size				
< 3	168(77.8)	48(22.2)	1.00	1.00
> or = 3	189(59.2)	130(40.8)	2.41(1.63–3.56)	3.16 (0.55–18.05)
Number of a live children				
No children	195(80.9)	46(19.1)	1.00	1.00
One child	61(65.6)	32(34.4)	2.22(1.30–3.80)	6.05 (0.18–12.97)
Two children	50(66.7)	25(33.3)	2.12(1.19–3.78)	0.54 (0.11–2.53)
Three and above	51(40.5)	75(59.5)	6.23(3.86–10.1)	4.85 (1.24–9.71) *
Autonomy to visit health facilities				
Yes	154(50.0)	154(50.0)	8.46(5.24–13.7)	**3.30 (1.45–6.92)** ^*^
No	203(89.4)	24(10.6)	1.00	1.00
Autonomy to visit friends and relatives				
Yes	83(44.1)	105(55.9)	4.75(3.23–6.99)	**3.95(1.28–12.17)** ^*^
No	274(79.0)	73(21.0)	1.00	1.00
With whom she lives				
With parents	126(90.6)	13(9.4)	1.00	1.00
With partner	31(23.7)	100(76.3)	31.3 (15.55–62.8)	**9.2 (2.84–13.60)** ^*^
Alone	159(80.3)	39(19.7)	2.38 (1.22–4.65)	5.28 (0.89–10.14)
Other	41(61.2)	26(38.8)	6.15 (2.89–13.05)	4.74 (0.42–5.29)
Exposure to mainstream media (Radio and/Television)				
Never	183(73.5)	66(26.5)	1.00	1.00
Rarely	103(65.2)	55(34.8)	1.48(0.96–2.28)	0.84 (0.25–2.79)
Sometimes (2–3 days/week)	44(78.6)	12(21.4)	0.76(0.376–1.52)	0.07 (0.01–1.32)
Almost every day	27(37.5)	45(62.5)	4.62(2.66–8.04)	5.9 (1.26–13.04) *
Discussed about SRH and services with their families				
Yes	53(36.6)	92(63.4)	6.14 (4.10–9.29)	9.36(3.44–17.46) *
No	304(77.9)	86(22.1)	1.00	1.00
Age of first sexual intercourse				
Before 15 years of age	30(88.2)	4(11.8)	1.00	1.00
15–18 years of age	62(43.4)	81(56.6)	9.8 (3.28–29.27)	5.63 (0.79–19.75)
After 18 years of age	65(47.4)	72(52.6)	8.31(2.78–24.85)	7.2 (1.51–13.40) *
Never had sexual intercourse	143(93.5)	10(6.5)	0.52 (0.15–1.79)	0.042 (0.012–1.86)
Do not remember	57(83.8)	11(16.2)	1.45 (0.42–4.94)	1.3 (0.13–4.93)
Materials available in proper format for disabled people at health facilities				
Yes	1(25.0)	3(75.0)	7.84 (0.81–76.23)	3.62 (0.21–19.30)
No	53(47.3)	59(53.7)	2.91(1.89–4.42)	2.41 (0.10–4.59)
I do not know	303(72.3)	116(27.7)	1.00	1.00

***Note***: ^*^p-value less than 0.05 and significantly associated, and 1.00: reference

**Abbreviations:** COR, Crude Odds Ratio; AOR, Adjusted Odds Ratio; CI, Confidence Interval.

## Discussion

This study was community based and 535 reproductive age (18–49 years) women with disabilities were participated. Of these, 44.3% of the women had physical/mobility disabilities followed by 35% (visual impairment) and 20.7%, hearing impairment. Since, this study aimed to assess the uptake of sexual and reproductive health services and associated factors, 33.27% (95%CI; 29.29–37.44) disabled women were using at least one SRH services in the last 12 months preceding to the survey. This study was in line with the study conducted in Arbaminch (33.7%) and Addis Ababa (31.1%), Ethiopia which were conducted to assess the use of family planning method alone [19, 34].

On the other side, the utilization of SRH service/s in the last 12 months in this study was lower than the study conducted in Ghana (70%) [35]. The study conducted in Ghana was among school age disabled students and assessed ever used of SRH services that might be the reason made high use. However, this study was higher than the study conducted in Gondar, Ethiopia (13.1%) [36]. The plausible reason for the disparities could be due to the difference of study participants, time gap and only one type of SHR service uptake that is family planning method was assessed in Gondar.

After controlling confounders, seven independent variables were significantly associated with SRH service utilization. Mothers who had three and above alive children were 4.85 times more likely to use at least one SRH service in the last 12 months preceding to the survey compared to those who had no child. Even if, no studies revealed that this factor as predictor to use SRH services among women with disabilities. However, it was consistent with the study conducted in Nepal, Ghana, and Malawi [37–39] among non-disabled women. The possible explanation could be mothers who had more children were married and reach the desire family size or want to limit the number of children compared to those who had no children, because most participants in this study used family planning methods from the five SRH services.

Living conditions were another factor affecting the use of SRH services. Mothers who lived with their sexual partner were 9.2 times more likely to use SRH services compared to those who lived with their parents. No studies clearly stated that living with partner is a predictor to use SRH services so far. Those who lived with their partners are more transparent and feel free to discuss reproductive health issues compared to those who lived with their parents. Social norms are the possible barriers that might be restricted to not make transparent communication with their parents, and result that consider themselves asexual. The study conducted in United States highlighted that people do not decide in women with disabilities health and they are regularly engaging in smoking and drinking alcohol because of discrimination [40].

The study done in Ghana showed that discussion about sexual actions with their daughters mentioned as protective strategy against irresponsible sexual behaviors [41], while those who live with partners have sexual intercourse experience and no hesitation to discuss sexual and reproductive health with their partner and neighbors. The study conducted in Northwest Ethiopia proved that those who experienced parental discussion on SRH issues had ever sexual intercourse and use some SRH services [42].

On the other side, those who had open discussion with their families about SRH services were 9.36 times more likely to uptake the service when compared with their counterparts. This finding is consistent with the study conducted in Kenya and Ethiopia among non-disabled adolescents [43, 44]. A systematic review in qualitative study revealed that communicative problem is a barrier to use health care services for women with disabilities [45]. Moreover, the study conducted in Nairobi, Kenya showed that parent-child communication is strongly associated with child’s safer sex practice, including using condom and delayed sexual debut [46]. The reason could be those who openly discussed with their family members would have sufficient awareness about the service benefits, where to be accessed, and comfort to use without any restriction. Lack of family support and low self-esteem may contribute to low utilization of the service.

Women who had full autonomy to visit health facilities, friends and relatives were the positive factors influenced SRH service utilization compared to those who had none. This result agrees with the studies conducted in Nepal and Nigeria [37, 47]. It has proved by the study conducts in Nigeria (here cited above) that autonomy plays a significant role in women’s use of SRH services which is independent of education and several other factors related to women’s status. In fact, that women who have great decision-making power can do what they want to do.

Reproductive-age disabled women who were exposed for Radio/Television every day were 5.9 times more likely to use at least one SRH service compared to those who never listened/watched. This finding is supported with the study done in Nepal [37]. The plausible explanation might be due to mainstream medias’ potential of disseminating health related information for the community who has limited educational attainment like these disabled women. The media plays a key role in amplifying awareness and encouraging the use of different health care services including SRH.

Those who started sexual intercourse after the age of 18 were 7.2 times more likely to use at least one SRH service in the last twelve months compared to those who started before 15. Previous studies have said nothing about this factor the probable reason for the association could be that those who are 18 and above years of age may use SRH service because they are married and not fears social discrimination (feel free) when they visit of health facilities.

### Limitations of the study

The data were self-reported and liable to recall bias and did not show the causal relationship due to the nature of cross-sectional study design. Our study also did not incorporate health care providers, family members and organizations working related to disabilities’ perspective were other limitations of this study.

### Implications

These findings focus on the needs for increased attention to sexual and reproductive health of reproductive age women with disabilities. The utilization of SRH health services in this study and along with the previous [19, 34, 36] suggest more could be done to improve SRH of women with disabilities. The exclusion of reproductive aged women with disabilities from SRH service interventions may increase the risk of adverse health outcomes, particularly has main impact on unplanned and unintended pregnancy, unsafe abortion, STIs infection including HIV/AIDS and infertility [10, 11]. Attending ANC, institutional delivery and postnatal care follow up are the components of SRH care services. Maternal disability is associated with increased the risk of pregnancy, delivery and postpartum complications [48]. Population based study conducted in United States highlighted that the odds of untended pregnancy had higher among women with disabilities compared to non-disabled women [49]. Low accessibility and utilization of SRH programs and information may result in poor preconception health as well [12].

Based on our findings, potential areas for action to improve the uptake of SRH care services among reproductive aged women with disabilities should include; women’s autonomy to decide and realize when they need to visit health facility, regular media exposure, open discussion with family about SRH and live with partner than parents.

Disabled women live with their partners were more likely by far to use SRH services compare to those who live with parents. Evidence revealed that women with disabilities often face stigma and discrimination from family in their reproductive health. Myths, misinformation and social norms are mentioned as reasons for individual belief and attitude [50].

Therefore, multifaceted understanding of reasons for low utilization of SRH services by involving other key-informants’ perspectives (health care providers and other stake holders) and consider in transforming social norms and beliefs about women with disabilities are the future research and intervention areas. Furthermore, all health facilities need to have sign language interpreter, decision support tool to decide and use SRH health care services.

## Conclusions

Reproductive age women with disabilities uptake of SRH services were the issue that needs action. Low uptake of SRH services has increased the risk of short-and long-term health outcomes. The concerned governmental and non-governmental organizations should make efforts to increase the uptake of SRH services and address factors associated with the outcome of interest variable. Additionally, the scientific community needs to investigate the barriers and facilitators to use SRH services from families, health care providers and other key informants’ perspective.

## Data Availability

The datasets used and analyzed during the current study are available from the corresponding author on reasonable request.

## Abbreviations

AOR: adjusted odds ratio
COR: crude odds ratio
HIV: human immunodeficiency virus
OR: odds ratio
PAC: post abortion care
SRH: sexual and reproductive health
STIs: Sexual transmitted infections
WHO: world health organizations
